# Association of hyperuricemia with disease severity in chronic hepatitis C patients

**DOI:** 10.1371/journal.pone.0207043

**Published:** 2018-11-05

**Authors:** Tyng-Yuan Jang, Ming-Lun Yeh, Ching-I Huang, Zu-Yau Lin, Shinn-Cherng Chen, Meng-Hsuan Hsieh, Chia-Yen Dai, Jee-Fu Huang, Chung-Feng Huang, Wan-Long Chuang, Ming-Lung Yu

**Affiliations:** 1 Hepatobiliary Division, Department of Internal Medicine, Kaohsiung Medical University Hospital, Kaohsiung Medical University, Kaohsiung, Taiwan; 2 Faculty of Internal Medicine, School of Medicine, College of Medicine, Kaohsiung Medical University, Kaohsiung, Taiwan; 3 Department of Occupational Medicine, Kaohsiung Medical University Hospital, Kaohsiung Medical University, Kaohsiung, Taiwan; 4 Department of Preventive Medicine, Kaohsiung Medical University Hospital, Kaohsiung Medical University, Kaohsiung, Taiwan; 5 Institute of Biomedical Sciences, National Sun Yat-Sen University, Kaohsiung, Taiwan; 6 College of Biological Science and Technology, National Chiao Tung University, Hsin-Chu, Taiwan; Nihon University School of Medicine, JAPAN

## Abstract

**Background/aims:**

Hepatitis C virus (HCV) infection is associated with extrahepatic manifestations such as metabolic abnormalities. The association between chronic hepatitis C (CHC) and uric acid levels has rarely been investigated. We aimed to evaluate the levels of serum uric acid in CHC patients.

**Methods:**

Three hundred and seventy-three histologically confirmed CHC patients who were scheduled to receive antiviral therapy were consecutively enrolled, and 746 age- and sex-matched uninfected controls were included for comparison. Hyperuricemia was defined as a uric acid level > 7 mg/dL in men and > 6.0 mg/dL in women.

**Results:**

Hyperuricemia was identified in 15.8% of the CHC patients. The uric acid levels did not differ between the CHC patients and the controls (5.54 ± 1.20 mg/dL vs. 5.45 ± 1.45 mg/dL, P = 0.3). Among the 373 CHC patients, the factors associated with hyperuricemia included body mass index (BMI) (OR/CI: 1.13/1.04–1.21, P = 0.003) and estimated glomerular filtration rate (eGFR) (OR/CI: 0.98/0.97–1.00, P = 0.02). Logistic regression analysis revealed that the factors associated with hyperuricemia in male patients included BMI (OR/CI: 1.12/1.05–1.30, P = 0.006) and advanced fibrosis (F3-4) (OR/CI: 0.27/0.09–0.83, P = 0.02), whereas the factors associated with hyperuricemia in female patients included eGFR (OR/CI: 0.97/0.95–0.99, P = 0.02) and diabetes (OR/CI: 3.03/1.11–8.25, P = 0.03). There was a significant decreasing trend of serum uric acid levels with the progression of fibrotic stages among male patients (6.21 ± 1.03 mg/dL 5.82 ± 1.16 mg/dL and 5.44 ± 1.28 mg/dL in stages F0-2, F3, and F4, respectively, trend P = 0.01).

**Conclusions:**

Hyperuricemia was inversely associated with liver disease severity in CHC male patients.

## Introduction

Hepatitis C virus (HCV) infection is one of the major etiologies of chronic liver disease worldwide, and it is estimated that >185 million people are anti-HCV seropositive globally[1]. Once chronic hepatitis C (CHC) has developed, it may progress to liver fibrosis, and 10% to 20% subjects develop cirrhosis or hepatocellular carcinoma within 10 to 30 years[2, 3]. HCV infection is also associated with extrahepatic manifestations including variable metabolic abnormalities, such as insulin resistance, metabolic syndrome and lipid derangement[4–6]. However, the association of CHC with serum uric acid has not been frequently investigated.

Uric acid is the end product of purine metabolism and is metabolized by the liver, muscles and the intestines[7]. Hyperuricemia is an indicator of many diseases such as cardiovascular disease[8], liver disease[9], and renal diseases[10]. The association of serum uric acid and liver disease has been more broadly explored in non-alcoholic fatty liver disease (NAFLD) and/or non-alcoholic steatohepatitis (NASH) patients, with inconsistent results obtained across studies[11–13]. Notably, less is known about the presentation of serum uric acid in CHC patients as compared to the general population. Moreover, its correlation to liver disease severity among CHC patients remains elusive. This study aimed to address the issue by comparing the uric acid levels between CHC patients and uninfected controls. Meanwhile, the level of uric acid was also studied within the well-characterized CHC cohort.

## Materials and methods

### Patients

Patients with CHC confirmed by biopsy scheduled to receive interferon-based antiviral treatment were consecutively recruited in a medical center in Taiwan from January 2006 to December 2010. CHC patients were excluded if they had the following conditions: a current or past history of alcohol abuse (≥20 g daily), co-infected with hepatitis B virus (HBV) and human immunodeficiency virus (HIV), and receiving anti-hyperuricemic agents. Another age- and sex-matched control group without HBV, HCV and HIV infections were recruited at a 1:2 ratio for comparison of the uric acid levels. Uric acid levels were tested before antiviral therapy in the CHC patients. For the controls, it was measured during the health check-up held in the Department of Preventive Medicine of the participating hospital. All patients were written informed consent before enrollment. The study was conducted according to the Declaration of Helsinki. The ethical committee of the Kaohsiung Medical University Hospital approved the study.

### Laboratory and histological analyses

Biochemical analyses including serum aspartate aminotransferase (AST) levels, alanine aminotransferase (ALT) levels and uric acid levels were measured on a multichannel autoanalyzer (Hitachi Inc, Tokyo, Japan). HCV antibodies (anti-HCV) were tested by a third-generation enzyme immunoassay (Abbott Laboratories, North Chicago, IL). Serum levels of HCV RNA were measured using the branched DNA assay (Versant HCV RNA 3.0, Bayer, Tarrytown, NJ; quantification limit: 615 IU/ml) for qualitative HCV RNA seropositivity. HCV genotypes were determined by the method described by Okamoto et al.[14].

Liver specimens were obtained from all CHC patients by liver biopsy within the six months prior to recruitment. The liver histology was staged as F0–4, according to the Metavir scoring system[15]. The association of uric acid with liver disease severity was judged and stratified by advanced fibrosis (F3-4) or no advanced fibrosis (F0-2)[16, 17]. The estimated glomerular filtration rate (eGFR) was calculated using the modification of diet in renal disease (MDRD) equation[18].

### Statistical analyses

The frequencies were compared between groups using the χ^2^ test with the Yates correction or Fisher’s exact test. Group means are presented as the mean (standard deviation) and were compared using analysis of variance and Student’s *t*-test or the nonparametric Mann-Whitney test, when appropriate. Hyperuricemia was defined as a uric acid level > 7 mg/dL in men and > 6.0 mg/dL in women[19]. Stepwise logistic regression analysis was applied to assess the factors associated with hyperuricemia by analyzing the co-variants with P values < 0.1 in the univariate analysis. Linear regression analysis was used to assess the factors correlated with serum uric acid levels. The Cochran-Armitage test was used to evaluate the trends of uric acid levels in patients with different disease severities. The statistical analyses were performed using the SPSS 20 statistical package (SPSS, Chicago, IL, USA). All statistical analyses were based on two-sided hypothesis tests with a significance level of p < 0.05.

## Results

### Patient characteristics

Three hundred and seventy-three histologically proven CHC patients and 746 age- and sex-matched uninfected controls were enrolled in the study. The mean age was 53.6 years (range: 18–80 years), and males accounted for 49.7% (n = 556) of the entire population. Compared to the uninfected individuals, CHC patients had significantly higher levels of AST, ALT and eGFR. The proportions of fibrotic stages F0-1, F2, F3 and F4 were 23.1% (n = 86), 42.9% (n = 160), 18.0% (n = 67) and 16.1% (n = 60), respectively, in CHC patients (Table 1).

**Table 1:** Characteristics of the 373chronic hepatitis C patients and 746 uninfected controls.

	All subjects (n = 1119)	Hepatitis C (n = 373)	Uninfected control (n = 746)	P value
Age (years, mean(SD))	53.6 (7.4)	53.6 (11.3)	53.6 (4.3)	0.90
Male, n (%)	556 (49.7)	183 (49.1)	373 (50.0)	0.77
BMI (kg/m^2^, mean (SD))	25.0 (3.8)	24.7 (3.6)	25.1 (3.9)	0.13
Uric acid (mg/dL)	5.48 (1.37)	5.54 (1.20)	5.45 (1.45)	0.30
Hyperuricemia, * n (%)	191 (17.1)	59 (15.8)	132 (17.7)	0.43
Diabetes, n (%)	153 (13.7)	57 (15.3)	96 (12.9)	0.27
AST (IU/L, mean (SD))	57.4 (58.1)	105.3 (55.5)	33.6 (42.6)	<0.001
ALT (IU/L, mean (SD))	73.8 (81.6)	153.5 (82.9)	33.9 (42.2)	<0.001
Serum creatinine ((mg/dL, mean (SD))	0.86 (0.16)	0.79 (0.21)	0.90 (0.12)	<0.001
Hemoglobin (g/dL, mean (SD))	14.1 (1.6)	14.0 (1.5)	14.1 (1.7)	0.24
Platelet count (x10^3^*u*/L, mean (SD))	217.3 (72.2)	167.2 (60.8)	242.3 (64.0)	<0.001
eGFR (ml/min/1.73m^2^, mean (SD))	80.7 (21.5)	100.8 (23.1)	70.6 (11.1)	<0.001
HCV RNA (log IU/mL, mean (SD))	-	5.34 (0.94)	-	-
HCV genotype 1/2/mixed1+2/others (n)		175/164/22/12	-	
Fibrosis, n (%)				
F0-1	-	86 (23.1)	-	-
F2	-	160 (42.9)	-	-
F3	-	67 (18.0)	-	-
F4	-	60 (16.1)	-	-

Note: SD: standard deviation; BMI: body mass index; AST: aspartate aminotransferase; ALT: alanine aminotransferase; eGFR: estimated glomerular filtration rate.

* Hyperuricemia defined as serum uric acid>7mg/dL in male and>6mg/dL in female

### Factors associated with hyperuricemia in the entire population

Among the study population, the mean uric acid level was 5.48 ±1.37 mg/dL, and the proportion of individuals with hyperuricemia was 17.1%. Univariate analysis revealed that the factors associated with hyperuricemia included male gender, higher BMI and lower eGFR. Multivariate analysis revealed that the factors associated with hyperuricemia included male gender (odds ratio [OR]/95% confidence intervals [CI]: 1.43/1.01–2.03, P = 0.04), BMI (OR/CI: 1.10/1.05–1.14, P < 0.001) and eGFR (OR/CI: 0.99/0.98–1.00, P = 0.01) (Table 2). The uric acid level did not differ between the CHC patients and the controls (5.54 ± 1.20 mg/dL vs.5.45 ± 1.45 mg/dL, P = 0.3) (Table 1)

**Table 2:** Factors associated with hyperuricemia of all subjects.

	Hyperuricemia * (n = 191, 17.1%)	Non-hyperuricemia (n = 928, 82.9%)	P value	Logistic regression analysis		
				OR	95% CI	*P* value
Age (years, mean(SD))	53.5 (7.6)	53.7 (7.4)	0.80			
Male, n (%)	116 (60.7)	440 (47.4)	0.001	1.43	1.01–2.03	0.04
BMI (kg/m^2^, mean (SD))	26.1 (3.8)	24.7 (3.7)	<0.001	1.10	1.05–1.14	<0.001
AST (IU/L, mean (SD))	59.5 (56.0)	57.0 (58.5)	0.57			
ALT (IU/L, mean (SD))	72.8 (70.8)	74.0 (83.6)	0.84			
HCV infection, n (%)	59 (30.9)	314 (33.8)	0.43			
eGFR (ml/min/1.73m^2^, mean (SD))	75.3 (20.0)	81.8 (21.6)	<0.001	0.99	0.98–1.00	0.01
Diabetes, n (%)	23 (12.0)	130 (14.0)	0.47			

Note: SD: standard deviation; BMI: body mass index; AST: aspartate aminotransferase; ALT: alanine aminotransferase; eGFR: estimated glomerular filtration rate. OR: odds ratio; CI: confidence intervals

* Hyperuricemia was defined as serum uric acid >7mg/dL in male and >6mg/dL in female

### Factors associated with hyperuricemia in CHC patients

Among the 373 CHC patients, univariate analysis revealed that the factors associated with hyperuricemia included higher BMI and lower eGFR. Multivariate analysis revealed that the factors associated with hyperuricemia included BMI (OR/CI: 1.13/1.04–1.21, P = 0.003) and eGFR (OR/CI: 0.98/0.97–1.00, P = 0.02) (Table 3). Male patients had a significantly higher serum uric acid level than female patients (6.03 ± 1.12 vs 5.08 ± 1.09 mg/dL, P < 0.001), although the proportion of individuals with hyperuricemia did not differ between genders. We further analyzed the uric acid levels in CHC patients according to gender. Among the 183 male patients, univariate analysis revealed that the factors associated with hyperuricemia included higher BMI and mild fibrotic stages (Table 4). Multivariate analysis showed that the factors associated with hyperuricemia in male patients included BMI (OR/CI: 1.12/1.05–1.30, P = 0.006) and advanced fibrosis (F3-4) (OR/CI: 0.27/0.09–0.83, P = 0.02) (Table 5).

**Table 3:** Factors associated with hyperuricemia in 373 chronic hepatitis C patients.

	Hyperuricemia * (n = 59, 15.8%)	Non-hyperuricemia (n = 314, 84.2%)	P value	Logistic regression analysis		
				OR	95% CI	*P* value
Age (years, mean(SD))	53.4 (12.4)	53.5 (11.2)	0.96			
Male, n (%)	28 (47.5%)	155 (49.4%)	0.79			
BMI (kg/m^2^, mean (SD))	26.2 (3.4)	24.4 (3.6)	0.002	1.13	1.04–1.21	0.003
AST (IU/L, mean (SD))	107.1 (49.5)	104.9 (56.8)	0.75			
ALT (IU/L, mean (SD))	152.5 (72.1)	153.7 (85.2)	0.67			
eGFR (ml/min/1.73m^2^, mean (SD))	92.3 (23.5)	102.7 (22.6)	0.002	0.98	0.97–1.00	0.02
Advanced fibrosis (F3-4), n (%)	19 (32.2%)	108 (34.3%)	0.74			
Diabetes, n (%)	12 (20.3%)	45 (14.3%)	0.24			
HCV RNA (log IU/mL, mean (SD))	5.43 (1.03)	5.32 (0.92)	0.46			
HCV genotype 1, n (%)	29 (49.2%)	168 (53.5%)	0.54			

Note: SD: standard deviation; BMI: body mass index; AST: aspartate aminotransferase; ALT: alanine aminotransferase; eGFR: estimated glomerular filtration rate; OR: odds ratio; CI: confidence intervals

* Hyperuricemia defined as serum uric acid >7mg/dL in male and >6mg/dL in female

**Table 4:** Univariate analysis of factors associated with hyperuricemia in chronic hepatitis C patients stratified by gender.

	Male		P value	Female		P value
	Hyperuricemia* (n = 28, 15.3%)	Non-hyperuricemia (n = 155, 84.7%)		Hyperuricemia* (n = 31, 16.3%)	Non-hyperuricemia (n = 159, 83.7%)	
Age (years, mean(SD))	46.9 (11.7)	51.6 (12.5)	0.06	59.4 (8.2)	55.4 (9.6)	0.02
BMI (kg/m^2^, mean (SD))	26.4 (3.6)	24.5 (3.4)	0.01	25.8 (3.6)	24.5 (3.7)	0.06
AST (IU/L, mean (SD))	109.4 (56.2)	96.6 (52.9)	0.27	105.4 (47.4)	112.9 (58.5)	0.44
ALT (IU/L, mean (SD))	169.3 (72.0)	157.5 (90.4)	0.45	132.0 (65.1)	150.9 (79.8)	0.16
eGFR (ml/min/1.73m^2^, mean (SD))	93.0 (25.3)	100.5 (23.5)	0.15	90.4 (24.0)	104.5 (21.5)	0.004
Advanced fibrosis (F3-4), n (%)	4 (14.3)	55 (35.5)	0.03	15 (48.4%)	53 (33.3%)	0.11
Diabetes, n (%)	4 (14.3%)	28 (18.1%)	0.63	8 (25.8%)	17 (10.7%)	0.02
HCV RNA (log IU/mL, mean (SD))	5.44 (1.03)	5.34 (0.91)	0.62	5.42 (1.05)	5.30 (0.93)	0.58
HCV genotype 1, n (%)	16 (57.1%)	92 (59.4%)	0.83	13 (41.9%)	76 (47.8%)	0.55

Note: SD: standard deviation; BMI: body mass index; AST: aspartate aminotransferase; ALT: alanine aminotransferase; eGFR: estimated glomerular filtration rate; OR: odds ratio; CI: confidence intervals

* Hyperuricemia defined as serum uric acid >7mg/dL in male and >6mg/dL in female

**Table 5:** Multivariate analysis of factors associated with hyperuricemia in chronic hepatitis C patients stratified by gender.

	OR	95% CI	P value
**Male**			
BMI (Per 1 kg/m^2^ increased)	1.12	1.05–1.30	0.006
Fibrotic stage			
F0-2	1		
F3-4	0.27	0.09–0.83	0.02
**Female**			
eGFR (Per 1 ml/min/1.73m^2^ increased)	0.97	0.95–0.99	0.02
Diabetes			
No	1		
Yes	3.03	1.11–8.25	0.03

Note: SD: standard deviation; BMI: body mass index; eGFR: estimated glomerular filtration rate; OR: odds ratio; CI: confidence intervals

**Fig 1:**
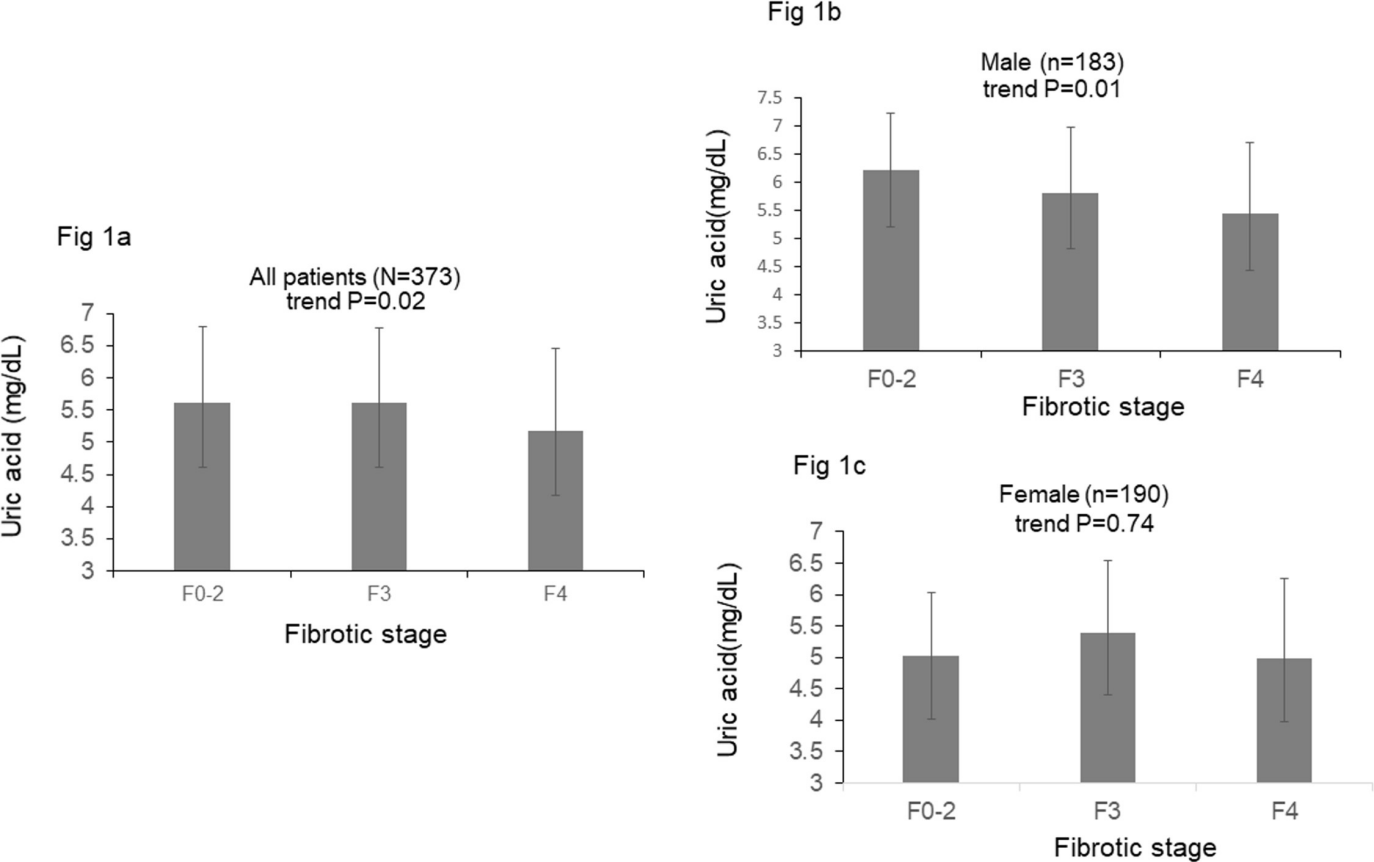
a. Serum uric acid levels in all patients with different fibrotic stages. b. Serum uric acid levels in male patients with different fibrotic stages. c. Serum uric acid levels in female patients with different fibrotic stages.

On the other hand, univariate analysis revealed that the factors associated with hyperuricemia in the 190 female patients included older age, lower eGFR and the presence of diabetes mellitus (DM) (Table 4). Multivariate analysis showed that the factors associated with hyperuricemia in females included eGFR (OR/CI: 0.97/0.95–0.99, P = 0.02) and DM (OR/CI: 3.03/1.11–8.25, P = 0.03) (Table 5). There was a significant decreasing trend of serum uric acid level with the progression of fibrotic stages (5.62 ± 1.18 mg/dL 5.61 ± 1.16 mg/dL and 5.17 ± 1.29 mg/dL in patients with F0-2, F3, and F4, respectively, trend P = 0.02). However, the significant association existed only in male patients (6.21 ± 1.03 mg/dL, 5.82 ± 1.16 mg/dL and 5.44 ± 1.28 mg/dL in male patients with F0-2, F3, and F4, respectively, trend P = 0.01) and not in female patients (5.02 ± 1.10 mg/dL, 5.40 ± 1.14 mg/dL and 4.98 ± 1.27 mg/dL in female patients F0-2, F3, and F4, respectively, trend P = 0.74) (Fig 1). Linear regression analysis also demonstrated that stages F3-4 were inversely correlated with uric acid levels only in male patients (β: -0.389; 95% CI: -0.718–0.060, P = 0.001) (Table 6).

**Table 6:** Stepwise linear regression analysis of factors associated with serum uric acid levels in chronic hepatitis C patients stratified by gender.

	B	Standard error	95% Confidence Interval for B		Beta	P value
**Male**						
Age	-0.017	0.007	-0.030	-0.004	-0.194	0.01
eGFR	-0.020	0.003	-0.026	-0.013	-0.414	<0.001
BMI	0.069	0.021	0.028	0.111	0.215	0.001
F3-4	-0.394	0.165	-0.720	-0.068	-0.165	0.02
**Female**						
eGFR	-0.011	0.003	-0.017	-0.004	-0.220	0.002
BMI	0.067	0.020	0.028	0.107	0.231	0.001

Note: Independent variables including age, eGFR, BMI, liver fibrosis and DM. BMI: body mass index; Odds ratio (OR) are for age (per 1 year increased), eGFR (per 1 ml/min/1.73m^2^ increased), BMI (per 1 kg/m^2^ increased), liver fibrosis (F3-4 vs F0-2)

## Discussion

In the current study, we demonstrated that the levels of serum uric acid were similar between CHC patients and the general population. The seroprevalence of hyperuricemia in the current survey was similar to that in a previous report from Taiwan[20]. Intriguingly, we noticed that there existed differences in uric acid levels in CHC patients according to gender and disease severity. Patients with the advanced liver disease had significantly lower uric acid levels. However, the clinical significance was only present in male patients. The more advanced the liver fibrosis, the lower the level of uric acid noted in male CHC patients.

Uric acid is the end product of purine metabolism, which is derived from endogenous and exogenous sources[21]. It is metabolized by the muscles, the intestines and the liver and is catalyzed by xanthine oxidase (XO). Approximately two-thirds of uric acid is excreted in urine, and the remaining one third is excreted in feces[7]. Female subjects have lower serum uric acid levels than male patients because of their higher plasma estrogen levels, which may contribute to a higher urate clearance in urine[22]. Several other risk factors have also been documented as being associated with hyperuricemia[23–26]. CHC patients are viewed as a special population with metabolic derangement. In the current study, we identified similar risk factors for hyperuricemia in CHC patients as in the general population[23, 26].

Hyperuricemia was associated with diabetes in particular among female patients. It might in part attribute to different physiologies between genders. Diabetes, being as a metabolic disorder, may enhance the impact of hyperuricemia in females who are with relatively low serum uric acid in nature [27]. Despite that the pathophysiology remains elusive, the current finding was in line with several previous studies [28–31].

Hyperuricemia was positively correlated to BMI, which also echoed the previous study[32]. Muscle volume is associated with hyperuricemia. Recently, sarcopenia is paid much attention in liver disease.[33, 34]. One of the risk populations is patients with catastrophic cirrhosis[34, 35]. Patients enrolled in the current study were planned to receive interferon-based therapy and all of them were with compensated liver disease. Although BMI could not completely represent the presence of sarcopenia, BMI did not differ between patients with or without advanced liver disease (data not shown). Moreover, only nine (2.4%) patients were lean (BMI<18.5 kg/m^2^) and none of them was cirrhotic. The issue and interference of sarcopenia in the current study may be less significant.

Previous studies that have addressed the relationship between liver diseases and uric acid have been largely restricted to patients with NAFLD or NASH. However, studies regarding the association of uric acid with disease severities have produced inconsistent results. Some reports demonstrated that hyperuricemia had no correlation with fibrotic stage in NASH or NAFLD patients[12, 13, 36, 37]. However, some other Asian studies have shown an inverse correlation between uric acid and liver fibrosis[11, 38]. On the other hand, the issue of serum uric acid level has been less often studied in CHC patients. Some have proposed its impact on antiviral treatment efficacy in the previous interferon era[39]. With respect to liver disease severity, Petta, S. et al. did not observe an association of uric acid level with liver fibrosis in Caucasians[40], which conflicts with our observation that there exists a dose-response relationship between liver fibrotic stages and uric acid levels in Chinese patients. Uric acid is metabolized by the liver, and it has been suggested that progressive liver damage may result in decreased serum uric acid production[11]. In addition, it has been suggested that cirrhotic patients have higher uric acid clearance than non-cirrhotic patients[41]. All the pathophysiological mechanisms support our finding. The percentage of individuals with hyperuricemia in an Italian cohort was only 7.5%, which may partially explain the insignificant differences in that study[40]. Further studies are needed to determine whether ethnic differences or diverse genetic backgrounds are responsible for the inconsistent results. Notably, uric acid metabolism was significantly different between male and female subjects in our study. When we separated patients by gender, uric acid levels had an inverse relationship with liver fibrosis only in male patients. The reason for this remains elusive. It has been suggested that testosterone has a negative impact on liver fibrosis[42]. Whether it enhances uric acid metabolism in male subjects[43] deserves further investigation.

The proportion of anti-hyperuricemic agents prescription in hyperuricemic subjects has been as low as 2.7% in Taiwan[44]. Only two biopsy-proven CHC patients received anti-hyperuricemic agents and were excluded before enrollment. The selection bias for interpreting the association of SUA with liver disease severity in this regard would be limited.

There were some limitations in the current study. Although we adjusted for certain critical confounders including DM and BMI, some documented risk factors of hyperuricemia including nutrition supplements and food intake, diuretics use, definite amounts of alcohol consumption and lifestyle were not taken into consideration precisely. CHC patients were preselected from a hospital-based cohort who were scheduled to receive interferon-based therapy in the era before direct-acting antivirals. Many of the CHC patients with the deteriorated renal function may have been excluded which may cause selection bias[45, 46]. CHC patients also had substantially lower body mass index. This may account for a lower creatinine level than was observed in the age- and sex-matched controls. We cannot exclude the possibility of altered uric acid levels in CHC patients provided by a similar renal function to that of the controls. The solution should be to recruit a large cohort of viremic patients without antiviral treatment from the community. However, that approach may be impractical, especially if histological data by invasive liver biopsy is needed. Notably, it held true that there was a negative correlation between serum uric acid levels and liver disease severity in CHC patients, regardless of the control group selection. In conclusion, hyperuricemia was inversely associated with liver disease severity in male CHC patients. As the metabolic derangements may be ameliorated after antiviral treatment [47, 48], further studies are warranted to clarify the potential alterations of serum uric acid levels after viral eradication.
